# Corneal astigmatism agreement between a swept-source ocular coherence tomography and Scheimpflug–Placido based optical biometers

**DOI:** 10.1007/s10792-025-03435-3

**Published:** 2025-03-06

**Authors:** Cameron McLintock, Samir Uprety, James McKelvie

**Affiliations:** 1 Department of Ophthalmology, Princess Alexandra Hospital, Brisbane, QLD Australia; 2 School of Medicine, University of Queensland, Brisbane, QLD Australia; 3 Vision for Life Institute, Brisbane, QLD Australia; 4 Department of Ophthalmology, University of Auckland, Auckland, New Zealand

**Keywords:** Corneal astigmatism, Keratometry, Anterion, Galilei G6

## Abstract

**Purpose:**

This prospective and comparative study assesses the agreement of anterior, posterior, and total corneal astigmatism measurements between swept-source optical coherence tomography (Anterion) and Scheimpflug cameras, Placido disc, and OCT-based tomography (Galilei G6).

**Methods:**

A total of 59 eyes of 59 patients were assessed using two optical biometers. Anterior, posterior, and total keratometry measurements were obtained, and flat (K1), steep (K2), astigmatic power, and J vectors (J_0_ and J_45_) were compared. Bland–Altman plots and intraclass correlation coefficients (ICC) were used to evaluate agreement and reliability between the devices.

**Results:**

Statistically significant differences were found for flat keratometry measurements of anterior corneal power (Galilei G6: 43.41 ± 1.78; Anterion: 43.32 ± 1.73) and total corneal power (Galilei G6: 42.41 ± 1.85; Anterion: 42.84 ± 1.81), as well as for steep keratometry of posterior corneal power (Galilei G6: − 6.41 ± 0.38; Anterion: − 6.31 ± 0.30) and total corneal power (Galilei G6: 43.69 ± 1.89; Anterion: 43.91 ± 1.89) (*p* < .05). In contrast, no statistically significant differences were found for the mean power vector components comparison between the devices. Agreement analysis showed significant proportional bias for cylindrical power and vector components (J_0_ and J_45_) of posterior and total astigmatism. No bias was observed with any of the anterior astigmatism and vector components. ICC showed showed relatively poor reliability (ICC < 0.5) between the device measurements moderate reliability for posterior corneal parameters.

**Conclusion:**

Anterior, posterior, and total astigmatism measurements between the Anterion and Galilei G6 are not interchangeable.

## Introduction

Epidemiological data shows more than 1 D of corneal astigmatism is present in nearly half of the eyes undergoing cataract surgery [1]. During surgery, toric intraocular lenses (IOLs) are implanted to minimize the astigmatic component of the postoperative refractive error. However, the efficacy of toric IOLs is highly dependent on the ability of ocular biometers to accurately measure the power and axis of the principal meridians. Conventionally, standard keratometry measured the anterior surface of the cornea and ignored posterior corneal astigmatism by assuming a fixed anterior/posterior curvature ratio. The measurement of posterior corneal power is now possible with the development of novel technologies and is essential to calculate total corneal power. Recent reports indicate that incorporating total corneal power into IOL calculations significantly improves the visual outcomes of cataract surgery [2–4].

The Anterion (Heidelberg Engineering GmbH) is a relatively new optical biometer based on swept-source optical coherence tomography (SS-OCT) that measures both anterior and posterior keratometry [5]. The Galilei G6 (Ziemer Ophthalmic Systems AG Port) is another widely used optical biometer that integrates dual rotating Scheimpflug cameras, Placido disc, and A-scan based on partial coherence interferometry to measure the anterior and posterior corneal surface and produce total corneal power using ray tracing techniques [6]. Only two studies [5, 7] have so far compared Anterion and Galilei G6 biometer parameters; however, none of the reports have directly compared both the magnitude and axis components of corneal astigmatism. Therefore, our study aimed to determine whether the measurements of anterior, posterior, and total astigmatism between these devices are interchangeable.

## Methods

This study was conducted in patients attending the outpatient ophthalmology clinic of Princess Alexandra Hospital, Brisbane, Australia. The study was prospective, cross-sectional and comparative in nature. Prior to invitation, all patients attending the clinics were informed about the purpose of the study. Written informed consent was obtained from all invited participants. The study adhered to the Declaration of Helsinki, and ethics approval (Approval reference no: LNR/2019/QMS/60356) was obtained from the Metro South Hospital Ethics Committee, Brisbane.

All patients went through a complete medical and ocular history review. A comprehensive ocular examination, including imaging of the posterior segment with OCT scans, was conducted for all patients. Each day prior to scanning, a mandatory calibration for each device was performed according to the manufacturer’s instructions. All biometry scans were performed by a single examiner. Both eyes were first scanned with the Anterion followed by the Galilei G6 with a maximum of three repeated scans were performed per eye until a quality scan was obtained. The quality of the scans was aligned with the device quality scoring metric of each manufacturer. Because of the built-in multiple measurement (least 3 to 5 repeated scans) that provides average results of the scans, we opted to use a single measurement for analysis. Only the right eye was randomly selected for inclusion in the analysis to minimize the effect of inter-eye correlation.

Healthy patients, including those with cataractous eyes, were included in the study. Given that corneal astigmatism > 0.5 D is only clinically important for toric intraocular lens selection, our selection criteria were set accordingly. Any ocular conditions that produced lower quality metrics or any corneal pathological conditions that may alter the corneal dimensions, such as corneal opacities, scars, dystrophies, ectasia like keratoconus, and a history of ocular surgery, were excluded from the study.

Both biometry scans produced keratometry: flat (K1) and steep (K2) measurements for anterior, posterior, and total corneal power. J_0_ and J_45_ vectors were then calculated for keratometry parameters from each participant using power vector analysis. To compare the overall astigmatism between the devices, the mean power vector components (J_0_ and J_45_) for each device were converted to mean astigmatic magnitude and axis form [8]. To compare individual astigmatism changes, first a difference in power vector between the devices was calculated as follows:

$$\begin{aligned} \Delta {\text{J}}_{0} & = {\text{J}}_{0} \;{\text{Anterion}} - {\text{J}}_{0} \;{\text{Galilei}} \\ \Delta {\text{J}}_{{{45}}} & = {\text{J}}_{{{45}}} \;{\text{Anterion}} - {\text{J}}_{{{45}}} \;{\text{Galilei}} \\ \end{aligned}$$

The individual difference in vector components was then converted to clinical notation (change in cylindrical power [negative] and axis) as follows:

$$\Delta \;{\text{Cylindrical}}\;{\text{power}} = - {2}\sqrt {{\Delta }J_{0}^{2} + {\Delta }J_{45}^{2} }$$

$$\Delta \;{\text{Axis}} = \frac{1}{2}\tan^{ - 1} \left( {\frac{{{\Delta }\;J_{0} }}{{{\Delta }\;J_{45} }}} \right)$$

### Statistical analysis

Microsoft Excel (Version 16.8) and GraphPad Prism (Version 10.1) were used for all statistical analyses. To generate polar plots of astigmatism analysis, the R program (Version 2023.12) was applied. The distribution of the datasets underwent normality checks with the Shapiro–Wilk test. According to the results of normality, parametric (paired two-tailed test) or non-parametric (Wilcoxon signed-rank) tests were applied to compare the mean or median values between the two devices. The agreement and reliability analysis were performed with the Bland–Altman method and intraclass correlation coefficient respectively. The 2.5% and 97.5% percentiles, representing the non-parametric limits of agreement, were estimated when the paired differences failed the normality assumption [9, 10]. To analyse the bias in the agreement analysis, a linear regression line was fitted to scatter plots of the Bland–Altman dataset, and the slope parameter was determined. A *p*-value < 0.05 was considered statistically significant. G*Power (Version 3.1.9.6) analysis resulted in 80% statistical power for the study sample.

## Results

This comparative and agreement study involved 59 eyes of 59 patients (25 male and 34 female) with a mean age of 59.63 ± 18 years.

The mean comparison of keratometry measurements and power vector components between the two devices is summarised in Table 1. Statistically significant differences between the devices were found for flat keratometry measurements of anterior (Galilei: Mean ± SD = 43.41 ± 1.78; Anterion: 43.32 ± 1.73), t(58) = 2.14, *p* = 0.037) and total corneal power (Galilei: 42.41 ± 1.85; Anterion: 42.84 ± 1.81), t(58) = − 6.50, *p* < 0.001) and for steep keratometry measurements of posterior cornea (Galilei: median (IQR): − 6.34 (− 6.56 to − 6.16); Anterion: − 6.26 (− 6.46 to − 6.16), Z = − 2.95, *p* = 0.003) and total corneal power (Galilei: 43.69 ± 1.89; Anterion: 43.91 ± 1.89), t(58) = − 3.13, *p* = 0.003). In contrast, no statistically significant differences were found for the mean power vector components comparison between the devices. The spherocylindrical form transformed from mean J vectors resulted in minimal change between the devices for both astigmatic power (range: 0.07–0.14 D) and axis (1–4°) (Table 2).

**Table 1:** Comparison of corneal biometrics between Galilei and Anterion

	Parameters	Galilei		Anterion		*p* value
		Mean ± SD	Median (IQR)	Mean ± SD	Median (IQR)	
Anterior	K1 (D)	43.41 ± 1.78	43.47 (42.09 to 44.49)	43.32 ± 1.73	43.48 (42.08 to 44.46)	0.037^a*^
	K2 (D)	44.46 ± 1.77	44.55 (43.38 to 45.53)	44.39 ± 1.78	44.39 (43.42 to 45.53)	0.155^a^
	*J*_0_	− 0.13 ± 0.49	− 0.30 (− 0.45 to 0.24)	− 0.16 ± 0.51	− 0.27 (− 0.46 to 0.09)	0.231^a^
	*J*_45_	0.03 ± 0.25	0.07 (− 0.16 to 0.22)	0.02 ± 0.27	0.03 (− 0.11 to 0.17)	0.828^b^
	*Fourier to Cylinder*	− *0.27 DC X 83°*		− *0.34 DC X 85°*		
Posterior	K1 (D)	− 6.00 ± 0.39	− 6.03 (− 6.25 to − 5.82)	− 6.04 ± 0.27	− 6.06 (− 6.18 to − 5.87)	0.271^a^
	K2 (D)	− 6.41 ± 0.38	− 6.34 (− 6.56 to − 6.16)	− 6.31 ± 0.30	− 6.26 (− 6.46 to − 6.16)	0.003^b*^
	*J*_0_	− 0.15 ± 0.17	− 0.13 (− 0.19 to − 0.08)	− 0.12 ± 0.06	− 0.12 (− 0.16 to − 0.08)	0.054^b^
	*J*_45_	0.00 ± 0.12	0.00 (− 0.05 to 0.02)	0.00 ± 0.05	0.00 (− 0.04 to 0.02)	0.369^b^
	*Fourier to Cylinder*	− *0.32 DC X 91°*		− *0.24 DC X 90°*		
Total	K1 (D)	42.41 ± 1.85	42.56 (41.42 to 43.43)	42.84 ± 1.81	42.97 (41.60 to 44.06)	< .001^a*^
	K2 (D)	43.69 ± 1.89	43.69 (42.41 to 44.89)	43.91 ± 1.89	43.89 (42.85 to 45.17)	0.003^a*^
	*J*_0_	− 0.13 ± 0.64	− 0.24 (− 0.47 to 0.34)	− 0.08 ± 0.55	− 0.2 (− 0.41 to 0.26)	0.210^b^
	*J*_45_	0.07 ± 0.35	0.06 (− 0.16 to 0.33)	0.03 ± 0.28	0.03 (− 0.10 to 0.18)	0.277^a^
	*Fourier to Cylinder*	− *0.30 DC X 76°*		− *0.16 DC X 80°*		

IQR: Interquartile range (Q1 to Q3)

^a^Paired t-test

^b^Wilcoxon’s signed-rank test

*Statistically significant: *p* < 0.05

**Table 2:** Agreement of corneal biometrics between Galilei and Anterion

	Parameters (Galilei–Anterion) (D)	Mean Difference	Median Difference	Lower LoA	Upper LoA	25% percentile	97.5% percentile	MAD	ICC
Anterior	Cylinder	0.01	0.005	− 0.55	0.58	− 0.50	0.48	0.21	0.92
	*J*_0_	0.04	0.03	− 0.45	0.53	− 0.30	0.53	0.15	0.93
	*J*_45_	0.01	0.02	− 0.47	0.48	− 0.26	0.32	0.14	0.72
Posterior	Cylinder	− 0.14	− 0.04	− 0.79	0.51	− 1.16	0.17	0.20	0.38
	*J*_0_	− 0.04	− 0.01	− 0.39	0.32	− 0.48	0.30	0.11	0.48
	*J*_45_	− 0.00	− 0.005	− 0.29	0.28	− 0.26	0.27	0.09	0.41
Total	Cylinder	− 0.20	− 0.03	− 1.48	1.07	− 2.0	0.63	0.42	0.74
	*J*_0_	− 0.06	− 0.01	− 0.82	0.70	− 1.01	0.61	0.25	0.88
	*J*_45_	0.05	− 0.02	− 0.60	0.69	− 0.65	0.90	0.21	0.63

*J*_0_ and *J*_45_ are power vector components

Mean difference and limit of agreement (LoA) are calculated based on the Bland–Altman analysis

MAD, Mean absolute difference

ICC, Interclass correlation coefficient

**Fig. 1:**
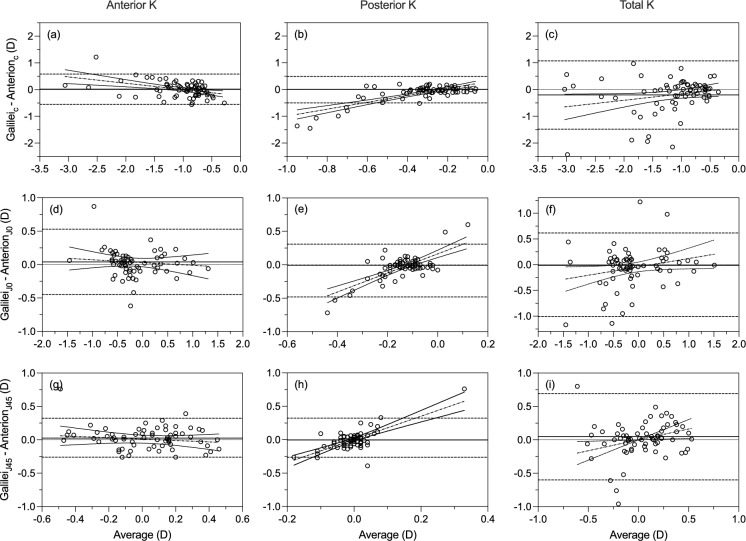
Bland–Altman plots for anterior K, posterior K, and posterior K astigmatic parameters: Astigmatic magnitude (**a**–**c**) and vector components J0 (**d**–**e**) and J45 (**g**–**i**). The solid line represents the mean difference between devices for figures **a**, **b**, **g**, and **i**, and the median difference for figures **c**, **d**, **e**, **f**, and **h**. Dashed lines indicate the upper and lower limits of agreement (LoA) for figures **a**, **b**, **g**, and **i**, and the 2.5th and 97.5th percentiles of the differences for figures **c**, **d**, **e**, **f**, and **h**, representing the non-parametric limits of agreement. The dotted-dashed line represents the regression line, along with the 95% confidence interval (solid curved line), assessing the bias

The agreement analysis between Galilei and Anterion for corneal cylindrical power and power vector components is summarised in Table 2. The mean cylindrical power difference between the devices for all keratometry (anterior, posterior, and total) measurements ranged from 0.01 to − 0.02 D. This is within the clinically acceptable difference (< 0.25 D). Among all the keratometry measurements, 95% limits of Agreement (LoA) of total keratometry cylindrical power were wider (− 1.48 to 1.07 D) compared to anterior and posterior measurements (Fig. 1c). The mean power vector difference between the devices ranged from 0.04 to − 0.06 D for J_0_ and 0 to 0.05 D for J_45_. Both the J vectors’ limit of agreement were found to have wider LoA (J_0_: − 0.82 to 0.70 D; J45: − 0.60 to 0.69 D) compared to anterior (J_0_: − 0.45 to 0.53 D; J45: − 0.48 to 0.14 D) and posterior power vectors (J_0_: − 0.39 to 0.32 D; J45: − 0.29 to 0.28 D) (Fig. 1f and i). The slope of the regression line showed a significant difference (*p* < 0.05) from 0 for cylindrical power and both J vectors of posterior and total astigmatism, indicating proportional biases between the device measurements (Fig. 1).

All parameters of anterior and total corneal measurements showed moderate (ICC = 0.5 to 0.8) to excellent reliability (ICC > 0.9) between the device measurements, except for posterior corneal parameters, which showed relatively poor reliability (ICC < 0.5) between the device measurements (Table 2).

**Fig. 2:**
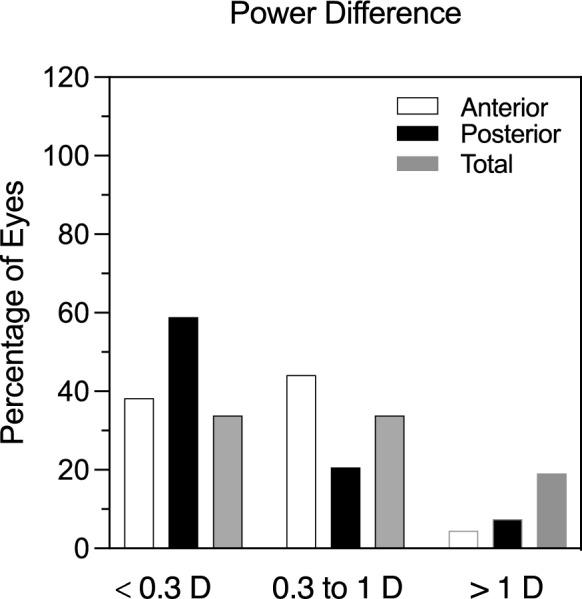
Frequency distribution of astigmatic difference in the anterior (AK), posterior (PK) and total keratometry (TK) group

The individual change in astigmatism can be obtained from vector transformation of individual change in cross cylindrical form. Figure 2 represents the distribution of the difference in corneal astigmatic magnitude between the devices. For anterior corneal astigmatism, 38% of eyes (26/59) showed corneal cylindrical power of less than 0.3 D, similarly, 44% of eyes (30/59) resulted in power differences in a range of 0.3 to 1 D, and only 4% of eyes showed differences greater than 1 D (3/59). For the posterior cornea, the majority (26%) (40/59) of the eyes showed corneal cylindrical power less than 0.3 D, and only 7% (5/59) of eyes showed corneal cylindrical power differences greater than 1 D. In contrast, 19% (13/59) of eyes showed corneal cylindrical power differences greater than 1 D.

**Fig. 3:**
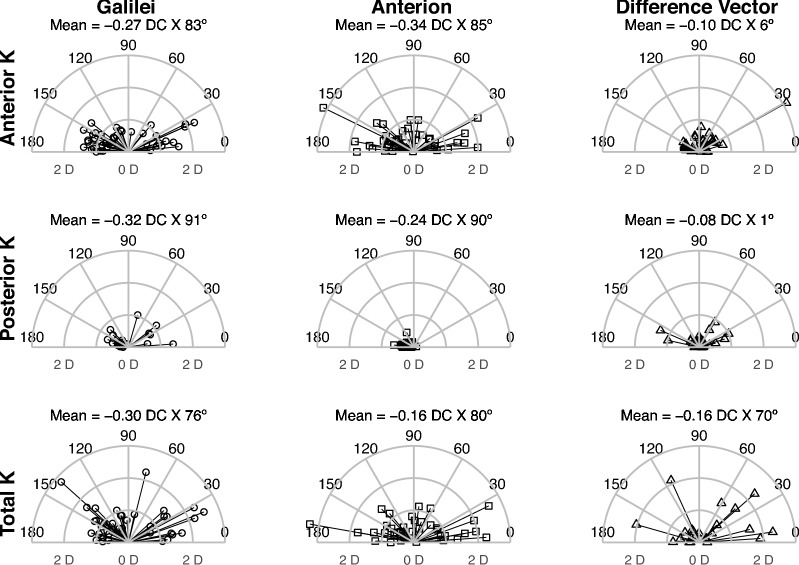
Polar plot of anterior, posterior, and total corneal astigmatism for the Galilei (circles) and Anterion (squares). Each data point represents the magnitude (radius) and axis (degree) of the negative correcting cylinder. The difference vector (triangle) indicates differences in corneal astigmatism between the two devices for each subject. The mean is obtained by converting the mean power vector to spherocylindrical form

**Fig. 4:**
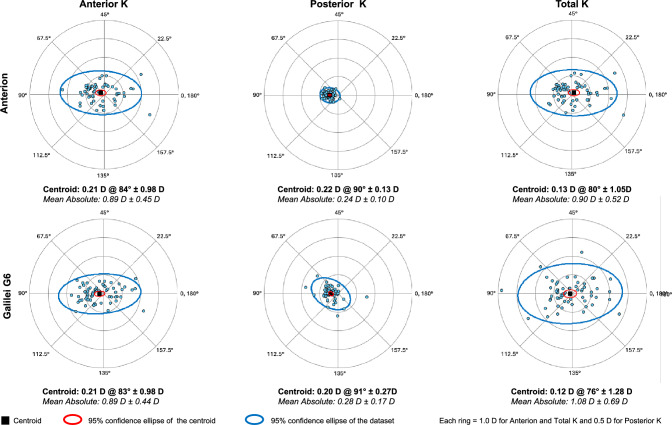
Double-angle plots visualizing the anterior, posterior, and total corneal astigmatism measured by the IOLMaster 700 and Anterion

Figure 3 shows vector analysis with double-angle plots and Fig. 4 shows a visual representation of individual astigmatism measurements, plotted as a vector for each device, along with the difference vector between the measurements, displayed in a polar plot. The magnitude and axis of the mean difference vector between the two devices were − 0.10 DC X 6° for anterior, − 0.06 DC X 1° for posterior, and − 0.16 DC X 70° for total keratometry measurements.

## Discussion

Our study found no statistically significant difference in anterior corneal astigmatic parameters and its power vector components, similar to previous studies that compared the Anterion with the Galilei G6 [5, 7]. The agreement analysis showed good reliability between the measurements of the two devices, with no significant bias. In comparison between device that compares SS-based optical biometers and the Scheimpflug analyzer, our data showed relatively lower mean than Jin et al. [11] (MD = − 0.15 D) and narrow LoA (− 1.11, 1.08) than Asawaworarit et al. [12].

The spherocylindrical transformation from the mean J vectors showed minimal mean differences in both magnitude and axis components of the astigmatism. However, individual differences in astigmatism magnitude revealed that more than 44% of eyes showed a power difference in the range of 0.3 D to 1 D, and 5% showed a difference greater than 1 D. This indicates that almost half of the eyes showed a power difference of > 0.3 D at the corneal plane, equivalent to 0.5 D at the IOL plane [13] With IOL power available in 0.5 D steps, the difference between the two devices could lead to clinically significant variations, particularly in toric IOL power calculations.

Interestingly, posterior corneal astigmatic parameters showed a statistically significant steep corneal surface and poor reliability for all astigmatic parameters. Although the agreement analysis showed the majority of the scatter data within the agreement limits, it clearly indicated a trend implying proportional bias. Similarly, Jin et al. also found comparable results, noting a significant difference in mean posterior corneal astigmatism parameters and poor reliability when comparing SS-based optical biometers and the Galilei G6 [11] Conversely, another study by Asawaworarit et al. [12] showed no difference in posterior astigmatism comparison. The mean spherocylindrical transformation showed minimal differences in both magnitude and axis components; however, individual transformations showed an astigmatic power difference of 0.3 D in the majority (60%) of the eyes. The discrepancy in the posterior astigmatic results may be associated with imaging technology. To measure the posterior cornea, the Anterion uses SS-OCT technology, on the other hand the Galilei G6 integrates a dual rotating Scheimpflug camera and Placido disc topography [6] Therefore, these variations in imaging technologies could explain the inconsistencies observed in posterior corneal astigmatism measurements, highlighting the need for careful consideration when interpreting results from different devices.

Total corneal measurements showed significant differences in both steep and flat corneal measurements. Our previous study [7] showed differences only in flat measurements, which may be due to the selection criteria used in the study (cylinder > 0.5 D). Agreement analysis revealed scattered data points with a wider LoA compared to anterior astigmatism, with proportional bias present in all astigmatic parameters. We found no significant difference in J vector components, and its transformation to spherocylindrical form showed minimal differences in magnitude (0.14 D) and a 4-degree discrepancy in the axis component of astigmatism. However, individual differences in magnitude revealed that 40% of eyes had a difference in the range of 0.3 D to 1 D, and 20% had a difference greater than 1 D. As both devices use ray tracing techniques to estimate total corneal power from anterior and posterior corneal measurements, discrepancies in total corneal astigmatism results may be due to differences in anterior and posterior measurements between the devices.

Our study focused on the agreement between the Galilei G6 and Anterion using single measurements per eye. Our data did not include enough samples with higher astigmatism to categorize astigmatism and compare the devices across different astigmatism levels. Future studies could explore comparisons, repeatability, and reproducibility to provide a more comprehensive understanding of these devices’ performance, as studies have reported discrepancies, particularly in moderate to high corneal cylinder measurements with the Galilei G6 [14]. Some effects in our study could have been overlooked because of insufficient power because of the variation in the astigmatic components’ effect size (Cohens d = 0.16 to 0.82). Future studies should be conducted as a multicentre study to account for the variability limited by a single-centre study and should have a bigger sample size to provide a large impact size for all variables.

Based on the results of this study, we recommend exercising caution against the interchangeable use of Anterion and Galilei G6 for corneal astigmatism measurements, especially in cases of higher degrees of astigmatism, due to the proportional bias observed. If total corneal measurements are used for the selection of toric lenses or refractive surgery, extra caution should be taken, considering the bias in posterior and total corneal measurements between the devices. To minimise error, multiple measurement methods or devices could be employed when high precision is required, or a single device could be used consistently for patient follow-ups to ensure comparability of measurements over time.

## Conclusions

In summary, we conclude that anterior, posterior, and total astigmatism measurements between the Anterion and Galilei G6 are not interchangeable. Future studies should include a larger range of corneal astigmatism data so that subgroup analysis can be performed to determine if bias exists in all subgroups. In addition, we compared data from healthy individuals only, but precise corneal astigmatism measurement is important in other conditions, such as contact lens assessment, planning of corneal refractive surgery, and monitoring the progression of corneal diseases like keratoconus. Therefore, future studies should consider agreement studies on such cohorts.

## Data Availability

The datasets are available upon reasonable request from the corresponding author.
